# Yellow Chaste Weed and Its Components, Apigenin and Galangin, Affect Proliferation and Oxidative Stress in Blue Light-Irradiated HaCaT Cells

**DOI:** 10.3390/nu14061217

**Published:** 2022-03-13

**Authors:** Jung Yoen Park, See-Hyoung Park, Sae Woong Oh, Kitae Kwon, Eunbi Yu, Seoyoung Choi, Seoyoun Yang, Su Bin Han, Kwangsun Jung, Minkyung Song, Jae Youl Cho, Jongsung Lee

**Affiliations:** 1 Molecular Dermatology Laboratory, Department of Integrative Biotechnology, College of Biotechnology and Bioengineering, Sungkyunkwan University, Suwon City 16419, Korea; maria0502@skku.edu (J.Y.P.); hanzeeoo@skku.edu (S.W.O.); wesdwe1@skku.edu (K.K.); yuebi95@skku.edu (E.Y.); csy2696@skku.edu (S.C.); chorim1004@skku.edu (S.Y.); subin816@skku.edu (S.B.H.); 2 Department of Bio and Chemical Engineering, Hongik University, Sejong City 30016, Korea; shpark74@hongik.ac.kr; 3 Biocosmetics Laboratory, TOUN28 Inc., Seongnam 13449, Korea; jks8835@toun28.com; 4 Integrative Research of T Cells Laboratory, Department of Integrative Biotechnology, College of Biotechnology and Bioengineering, Sungkyunkwan University, Suwon City 16419, Korea; 5 Molecular Immunology Laboratory, Department of Integrative Biotechnology, College of Biotechnology and Bioengineering, Sungkyunkwan University, Suwon City 16419, Korea

**Keywords:** blue light, keratinocytes, TRPV1, calcium influx, ROS, clusterin, FoxO3a, cell proliferation, apoptosis

## Abstract

While harmful effects of blue light on skin cells have been recently reported, there are few studies regarding natural products that alleviate its negative effects. Therefore, we investigated ameliorating effects of yellow chaste weed (YCW) (*Helichrysum arenarium*) extract and its components, apigenin and galangin, on blue light-irradiated HaCaT cells. In this study, we found that YCW extract improved the reduced proliferation of HaCaT cells induced by blue light-irradiation and reduced blue light-induced production of reactive oxygen species (ROS) levels. We also found that apigenin and galangin, the main components of YCW extract, showed the same activities as YCW extract. In experiments examining molecular mechanisms of YCW extract and its components such as apigenin and galangin, they all reduced expression of transient receptor potential vanilloid member 1 (TRPV1), its phosphorylation, and calcium ion (Ca^2+^) influx induced by blue light irradiation. In addition, apigenin and galangin regulated phosphorylation of mitogen-activated protein kinases (MAPKs). They also reduced phosphorylation of mammalian sterile 20-like kinase-1/2 (MST-1/2), inducing phosphorylation of Akt (protein kinase B), one downstream molecule of MST-1/2. Moreover, apigenin and galangin promoted translocation of Forkhead box O3 (FoxO3a) from the nucleus to the cytosol by phosphorylating FoxO3a. Besides, apigenin and galangin interrupted blue light influences on expression of nuclear and secretory clusterin. Namely, they attenuated both upregulation of nuclear clusterin and downregulation of secretory clusterin induced by blue light irradiation. We also found that they downregulated apoptotic protein Bcl-2 associated X protein (Bax) and conversely upregulated anti-apoptotic protein B-cell lymphoma 2 (Bcl-2). Collectively, these findings indicate that YCW extract and its components, apigenin and galangin, antagonize the blue light-induced damage to the keratinocytes by regulating TRPV1/clusterin/FoxO3a and MAPK signaling.

## 1. Introduction

The skin is located at the outermost part of the human body, protecting us from a variety of external factors, including sun light, pollutants, and stresses. These various external factors induce many physiological changes in skin, such as aging, pigmentation/vitiligo, skin inflammation, and hair loss. Therefore, identifying the signal transduction caused by the external factors and developing treatments targeting them are important challenges in skin research [1,2,3,4].

Sunlight, one of the biggest factors affecting the skin, is largely composed of ultraviolet (UV), visible and infrared light, depending on energy and wavelength [5,6]. Among the various wavelengths, blue light (380–500 nm), also called high-energy visible (HEV) light, has especially received attention because of the increased use of electronic devices such as smartphones, laptops, computers, and TVs in everyday life [7,8]. Excessive exposure to blue light has been reported to induce several damages to skin cells, including oxidative stress, apoptosis, and reduced proliferation potential [9,10]. In particular, blue light induces several damages to the epidermis, including DNA damage and oxidative stress [11,12,13].

The transient receptor potential vanilloid member 1 (TRPV1) channel is a submember of the TRP cation channel family, expressed in kidney cells, bronchial epithelial cells, primary sensory neurons, and keratinocytes. TRPV1 is a non-selective cation receptor and responds to capsaicin. TRPV1 is activated by heat (>43 °C), low pH, ultraviolet, and blue light. Activated TRPV1 can also induce Ca^2+^ influx in keratinocyte and mediate multiple signaling pathways. Blue light has been recently reported to upregulate and activate TRPV1, leading to ROS production and reduced cell proliferation [14]. In addition, in our previous report, we demonstrated that the effects of blue light on keratinocyte cell proliferation are mediated by upregulating TRPV1, a negative regulator of EGFR-FoxO3a signaling [10]. Blue light-induced production of ROS and TNF-α is also mediated through increased calcium influx via TRPV1 activation.

Calcium ions (Ca^2+^) act as a secondary messenger for controlling various aspects of cell functions such as differentiation in keratinocytes and barrier homeostasis [15,16,17]. Calcium-sensing receptor and calcium-permeable channels expressed in skin could regulate various functions in skin barrier homeostasis [18,19,20,21,22]. Clusterin expression is also controlled by intracellular Ca^2+^ influx [23,24]. Clusterin, a glycoprotein which is highly expressed and ubiquitously synthesized in the body, can be characterized into two isoforms: anti-apoptotic secretory CLU (sCLU) or apoptotic nuclear CLU (nCLU). These two forms of clusterin are controlled differently by Ca^2+^ [25,26].

The Forkhead box O (FoxO) transcription factors include FoxO1, FoxO3a, FoxO4, and FoxO6 [27]. Notably, FoxO3a is well known to mediate cellular events, such as apoptosis, proliferation, aging, and longevity in various types of cells [27,28,29,30,31]. FoxO3a is the downstream of Akt, also known as protein kinase B (PKB), thereby the phosphorylation of the FoxO3a is modulated by Akt [32,33,34]. Phosphorylated FoxO3a by Akt induces binding with 14-3-3 protein, which moves FoxO3a from the nucleus to the cytoplasm [35]. Furthermore, in the presence of oxidative stress, FoxO3a is controlled by c-Jun N-terminal kinase (JNK)/mammalian sterile 20-like kinase 1 (MST 1) signaling pathways [36]. As JNK is activated by oxidative stress, the phosphorylation of 14-3-3 weakens the coherence with the FoxO3a, and subsequently isolated FoxO3a moves from cytoplasm to the nucleus, leading to the transactivation of the target genes [37].

In the aspect of preventing the adverse effects of blue light on the skin, there is a need to develop blue light-antagonizing agents, using natural or natural-derived ingredients or organic ingredients. In this study, we examined the possibility of yellow chaste weed (*Helichrysum arenarium*) and its components as agents to improve blue light-induced damage. The yellow chaste weed has been used as a traditional medicine since ancient times [38]. Apigenin (4′,5,7-trihydroxyflavone) and galangin (3,5,7-trihydroxy flavones) are the two main flavonoids of yellow chaste weed [39,40]. They have been reported to possess anti-cancer, antioxidant, and anti-inflammatory properties [41,42,43,44,45,46,47,48,49,50]. Although the biological properties of apigenin and galangin have already been extensively studied, it remains unclear how they affect blue light-irradiated HaCaT cells.

Therefore, in this study, we investigated antagonizing activities of yellow chaste weed and its components against blue light and its mechanisms of action in human keratinocytes, HaCaT cells.

## 2. Materials and Methods

### 2.1. Cell Culture and Materials

HaCaT cells, a human keratinocyte cell line, were obtained from ATCC (Manassas, VA, USA). The cells were cultured in Dulbecco’s modified Eagle’s medium (DMEM; SH30243.01, Hyclone, Logan, UT, USA) supplemented with 10% fetal bovine serum (FBS) and 1% of antibiotics (penicillin and streptomycin), at 37 °C in a humidified incubator (5% CO^2^). The medium was changed every three days until the cells grew 70–80% confluence.

All types of antibodies were used in Western blot analysis. Antibodies for TRPV1 (1:1000 dilution, PA1-29421) and p-TRPV1 (Ser502) (1:1000 dilution, PA5-64860) were purchased from Invitrogen (Invitrogen, Waltham, MA, USA). Antibodies for JNK (1:2000 dilution, sc-572), p-JNK (1:1000 dilution, sc-6254), ERK 1/2 (1:2000, sc-292838), p-ERK 1/2 (Tyr 204) (1:2000 dilution, sc-101761), p38 MAPK (1:1000 dilution, sc-535), Lamin B1 (C-5) (1:500 dilution, sc-365962), Clusterin (1:1000 dilution, sc-166907), Clusterin-α (1:1000 dilution, sc-5289), Bcl-2 (1:1000 dilution, sc-7382), and Bax (1:1000 dilution, sc-7480) were purchased from Santa Cruz Biotechnology (Santa Cruz, Dallas, TX, USA). Antibodies for FoxO3a (1:2000 dilution, 2497S), p-FoxO3a (Ser253) (1:1000 dilution, 9466S), p-p38 MAPK (1:2000 dilution, 9216S), Akt (1:1000 dilution, 9272S), and p-Akt (Ser473) (1:1000 dilution, 12694S) were purchased from Cell Signaling Technology (CST, Danvers, MA, USA). Antibodies for Clusterin (1:1000 dilution, ab69644), α-tubulin (1:10,000 dilution, ab7291), and goat anti-mouse IgG (1:4000 dilution, Alexa fluor 488, ab150117) antibodies were ac-quired from Abcam (Abcam, Cambridge, UK). Antibodies for β-actin (1:4000 dilution, A5316), anti-rabbit immunoglobulin G (IgG) (1:4000 dilution, A0545), and anti-mouse IgG (1:4000 dilution, A9044) were purchased from Sigma–Aldrich (Sigma–Aldrich, St. Louis, MO, USA). Antibodies for p-MST 1 (Thr183)/MST 2 (Thr180) (1:1000 dilution, bs-3294R) were purchased from Bioss (Bioss Inc., Wo-burn, MA, USA).

### 2.2. YCW Extract, Apigenin and Galangin Pretreatment, and Blue Light Irradiation

YCW extract was obtained from Luvama Biolab Co., Ltd. (Seongnam, Korea). The powder of the extract was dissolved in dimethyl sulfoxide (DMSO) (472301, Sigma–Aldrich, St. Louis, MO, USA) for the experiments. Apigenin (SMB00702, Sigma–Aldrich, St. Louis, MO, USA), and galangin (sc-235240, Santa Cruz Biotechnology, Dallas, Texas, USA) were dissolved in DMSO. On the first day, HaCaT cells were treated with various concentrations of YCW extract (0.01, 0.05, 0.1%) or apigenin (10, 20, 30 μM) or galangin (1, 10, 20 μM) or vehicle (DMSO) for 24 h in phenol red-free culture medium (PRFCM) before blue light irradiation. On the second day, the culture media were removed, and the cells were incubated with fresh PRFCM. Then, the cells were irradiated with blue light with a photo-reactor (CCP-4V, Luzchem, Ottawa, ON, Canada) that had an emission peak between 470–480 nm. The power density of LED blue light equipped in photoreactor was 76 W/m^2^, and it was exposed for 30 min. The dose (76 W × 30 min/m^2^ = 13.68 J/cm^2^) of blue light irradiation used is equivalent to those received by the skin during summer after proximately 1500 s of sun exposure. After the first blue light irradiation (13.68 J/cm^2^), cells were incubated in the presence of the indicated concentration of YCW extract, apigenin, and galangin for 24 h at 37 °C. On the third day, the same process as on the second day was repeated. In order to consider temperature as a control variable, the control group was also left in the dark at room temperature, while the experimental groups were irradiated.

### 2.3. Cell Counting Kit-8 Assay for Cytotoxic Analysis

The cytotoxicity effect of apigenin and galangin on HaCaT cells were measured by cell counting kit-8 assay (CCK-8; CK04-11, Dojindo, Japan). Cells were cultured in 6-well plates. After changing culture media, the cells were incubated for 24 h with apigenin and galangin. These processes were repeated twice more to make a total treatment time of 72 h. After incubation, cells were washed with PBS and the media was changed to PRFCM. Next, the CCK-8 reagent (8 µL/well) was added to cells and incubated for 2 h at 37 °C. Then, the equal amount of supernatant (150 µL/well) was taken to measure the absorbance at 450 nm with microplate reader (Synergy HTX Multi-Mode Reader, BioTek, Winooski, VT, USA).

### 2.4. CellTiter-Glo^®^ 2.0 Assay for Cell Proliferation Analysis

Cells were cultured on 96-well opaque wall. After 4 days of rough processing (24 h pretreatment and blue light irradiation × 2 days), plates were placed at room temperature for 30 min to equilibrate the plate and its contents to optimal temperature. The CellTiter-Glo^®^ 2.0 reagent (G9242, Promega, Madison, WI, USA) equal to the volume of cell culture medium was presented in each well (100 µL). All the contents were mixed for 2 min on orbital shaker to induce cell lysis. Cells were incubated at room temperature for 10 min with light-blocked to stabilize the luminescent signal. Luminescence was recorded using a microplate reader (integration time of 0.5 s per well).

### 2.5. BrdU ELISA for Cell Proliferation Analysis

The cells were plated on 96 well black-wall/clear-bottom plates and proceeded 4 days repeated processing experiment. After the experiment, the cell proliferation was determined using the Cell Proliferation ELISA BrdU (colorimetric) kit (11647229001, Roche, Basel, Switzerland) in accordance with the manufacturer’s instructions.

### 2.6. EdU Incorporation Imaging for Cell Proliferation Analysis

Click-iT^®^ EdU Imaging Kits (C10337, Invitrogen, Waltham, MA, USA) were used to examine the cell proliferation. Cells were cultured in confocal plates. Apigenin 30 μM, galangin 20 μM, and vehicle (DMSO) were pretreated for 24 h on the first day. The next day, PRFCM were changed, cells were irradiated blue light (13.68 J/cm^2^) and treated with indicated concentrations of substances. On the third day, same processes as on the second day were proceeded. Twelve hours after blue light (13.68 J/cm^2^) exposure, cells replaced half of the media with an equal volume of EdU labeling solution (final concentration of 10 μM) and incubated for 12 h at 37 °C. From this time on, cells were washed three times with 1 mL of PBS with 0.1% Tween-20 (PBST) before every steps. After incubation, cells were fixed with 1 mL of 4% formaldehyde in PBS for 15 min at room temperature. Fixative was removed, 1 mL of 0.1% Triton X^®^-100 in PBS to permeabilize cells was added, and then incubated for 20 min at room temperature. After that, cells were blocked with 500 µL of 3% BSA in PBS for 1 h at room temperature. Next, cells were added with 200 µL of Click-iT^®^ reaction cocktail and incubated for 30 min at room temperature in the dark. After EdU detection, DNA staining was carried out by adding 200 µL of Hoechst 33342 (Invitrogen, Waltham, MA, USA) in PBS, and incubated for 10 min at room temperature in the dark. Thereafter, cells were washed three times with PBS and wet mounted with PBS for slide preparation. Finally, the cells were observed using an LSM 700-laser scanning confocal microscope (Zeiss, Jena, Germany). To measure the intensity of immunofluorescence, images were taken on the same laser power and the mean value of signals was evaluated. Signals from the images were measured by using ZEN 2012 Blue (Zeiss, Jena, Germany) and ImageJ software (National Institutes of Health, Bethesda, MD, USA).

### 2.7. Flow Cytometry Analysis for Apoptosis

Cells were cultured in 60-mm plates and after 4 days of rough processing (24 h pretreatment and blue light irradiation × 2 days), suspended media were first collected, and cells were washed with 1 mL of PBS and collected on 15-mL tubes. Tubes were then centrifuged at 21,209× *g* for 3 min. Cells were harvested with 500 µL of trypsin and centrifuged at 1500 rpm for 3 min and removed supernatant. Cell pellets were resuspended with 100 µL of 1X annexin binding buffer with diethyl pyrocarbonate-treated double distilled water (DEPC-DW; C-9030, Bioneer, Daejeon, Korea). Cells were then stained with 100 µg/mL of propidium iodide (PI) and 5 µL of FITC-Annexin V using Dead Cell Apoptosis Kit with Annexin V FITC and PI, for flow cytometry (V13242, Invitrogen, Waltham, MA, USA) and incubated for 15 min at room temperature in the dark. After the incubation, mix gently with 400 µL of 1X annexin binding buffer and keep the samples on ice. The stained cells were counted at least 10,000 cells and analyzed by flow cytometry (CytoFLEX, Beckman Coulter, Brea, CA, USA).

### 2.8. Fluo-4 NW Calcium Assay for Intracellular Calcium Influx Analysis

Cells were cultured in 96-well black wall/clear bottom plates. Cells were pretreated with yellow chaste weed extract 0.1% or apigenin (10, 20, 30 μM) or galangin (1, 10, 20 μM) or vehicle (DMSO) for 1 h in PRFCM. Next, 100 µL of 1X Fluo-4 NW dye loading solution (F36205, Invitrogen, Waltham, MA, USA)/well were added to the cells and plates were incubated in the dark at 37 °C for 30 min, then at room temperature for an additional 30 min. After removing dye loading solution, 100 µL of assay buffer (1X HBSS, 20 mM HEPES) were added to cells and blue light (13.68 J/cm^2^) were irradiated in plates except for control group. Then, cells were treated with indicated concentrations of yellow chaste weed extract or apigenin or galangin or vehicle (DMSO) dissolved in assay buffer. Fluorescence was measured using a microplate reader (excitation, 494 nm; emission, 516 nm).

### 2.9. DCF-DA Fluorescence Assay for ROS Production Analysis

The cellular ROS were measured by 2′,7′-dichlorofluorescin diacetate (DCF-DA) assay kit (ab113851, Abcam, Cambridge, UK). The cells were plated on 96-well black-wall/clear-bottom plates. Cells were pretreated with apigenin (10, 20, 30 μM), galangin (1, 10, 20 μM), and vehicle (DMSO) for 24 h with culture media. They were rinsed twice in PBS and treated with 20 μM DCF-DA (freshly diluted in PBS) for 30 min at 37 °C with the light blocked. After incubation, they were replaced with PBS and blue light (30 min) were irradiated. Fluorescence signals were detected using a microplate reader (excitation, 485 nm: emission, 535 nm).

### 2.10. Western Blotting

Cells were cultured in 60-mm plates and treated with indicated concentrations and incubation time of yellow chaste weed, apigenin, and galangin. After that, cells were har-vested and total proteins were extracted from the cells using RIPA buffer (9806S, Cell Signaling Technology, MA, USA), containing protease inhibitor cocktail (5872S, Cell Signaling Technology, Danvers, MA, USA). Nuclear and cytoplasmic fractions were extracted using NE-PER Nuclear and Cytoplasmic Extraction Reagents (78833, Thermo Fisher Scientific, Waltham, MA, USA), following manufacturer’s instructions. Pierce^TM^ BCA Protein Assay Kit (23227, Thermo Fisher Scientific, Waltham, MA, USA) was used to quantify ac-curate protein sample concentrations. Protein samples to be loaded were mixed with 4X sample buffer (161-1747, Bio-Rad, Hercules, CA, USA) and heated at 95 °C for 5 min. The protein samples were separated by sodium dodecyl sulfate-polyacrylamide gel electrophoresis (SDS-PAGE) and transferred onto polyvinylidene fluoride (PVDF) membrane (162-0177, Bio-Rad, Hercules, CA, USA). After transfer, the proteins were dyed with Ponceau S solution (P7170, Sigma–Aldrich, St. Louis, MO, USA), and sufficiently washed with Tris-buffered saline and 0.1% Tween-20 (TBST). Membranes were blocked for 2 h using 2% BSA and were incubated overnight with the primary antibodies at 4 °C. Then, the membranes were incubated for more than 1 h with the secondary antibodies at room temperature. Protein bands were visualized by Pierce^TM^ ECL Western blotting substrate (23227, Thermo Fisher Scientific, Waltham, MA, USA).

### 2.11. Statistical Analysis

The data are expressed as the mean ± standard error of the mean (SEM). Analyses of differences between two groups were performed using Student’s *t*-test. The comparison between multiple groups was performed using one-way analysis of variance (ANOVA), followed by the Tukey’s multiple-comparison test, for which the GraphPad Prism (5.0) (GraphPad, La Jolla, CA, USA) software was used. Statistical significance was considered when the *p*-value was less than 0.05.

## 3. Results

### 3.1. Effect of YCW Extract on the Blue Light Irradiated HaCaT Cells

**Figure 1:**
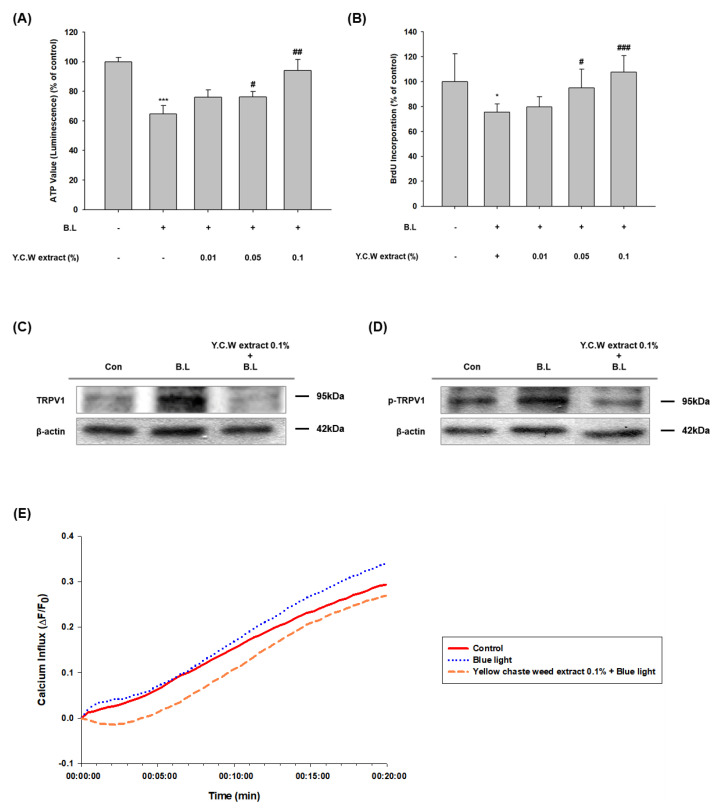
YCW extract increased cell proliferation via calcium dependent-TRPV1 signaling pathways in blue light irradiated HaCaT cells. (**A**) CellTiter-Glo^®^ 2.0 assay of YCW extract (0.01–0.1%) pretreated with blue light-irradiated HaCaT cells. YCW extract was pretreated 24 h before irradiation of blue light (13.68 J/cm^2^), and the same process was repeated on the next day. After 24 h incubation, an equal volume of CellTiter-Glo reagent was added to the cells. (**B**) Cell proliferation effect of YCW extract (0.01–0.1%) on blue light-irradiated HaCaT cells was measured using BrdU ELISA assay. Data are presented as the mean ± SEM of four independent experiments. Statistical significance of differences among the groups was assessed by one-way analysis of variance (ANOVA), followed by Tukey’s multiple-comparison test, using the GraphPad Prism 5 software. * *p* < 0.05 vs. control group. * *p* < 0.05 vs. control, *** *p* < 0.005 vs. control, # *p* < 0.05 vs. blue light-irradiated group (B.L), ## *p* < 0.01 vs. B.L. ### *p* < 0.005 vs. B.L. (**C**) TRPV1 expression levels were determined by Western blotting at 24 h incubation after, pretreatment with YCW extract (0.1%) for and 2-day repetitive blue light irradiation (30 min, 76 W/m^2^). (**D**) Phosphorylated-TRPV1 expression levels were determined by Western blotting at 90 min incubation after, 1 h YCW extract (0.1%) pretreatment and blue light irradiation (30 min, 76 W/m^2^). The total proteins were extracted from the cells immediately after experimental conditions, and β-actin was used as a loading control. (**E**) Ca^2+^ influx changes of blue light and with Y.C.W extract (0.1%) pretreatment by Fluo-4 NW assay. Cells were 1-h pretreated with YCW extract (0.1 %) and irradiated with blue light (10 min, 76 W/m^2^). After the irradiation, fluorescence intensities were measured immediately over a certain period.

To examine the effects of YCW extract on the proliferation of HaCaT cells, cells were treated with various concentrations (0.01, 0.05, 0.1%) of YCW extract. When blue light-exposed HaCaT cells were treated with YCW extract, the proliferation was significantly increased (Figure 1A,B). The effect of blue light on the activation of TRPV1 in HaCaT cells was demonstrated by our previous study [10]. Next, we investigated the influence of YCW extract on expression and phosphorylation of TRPV1. After blue light irradiation, the expression of TRPV1 and phospho-TRPV1 increased apparently (Figure 1C,D). However, YCW extract-pretreated group has shown downregulation of TRPV1 and reduction of its phosphorylation (Figure 1C,D). TRPV1 is a non-selective cation channel and the activation of TRPV1 elevates calcium influx [51]. To evaluate the inhibition effect of YCW extract on Ca^2+^ influx, cells were pretreated with YCW extract 0.1% for 1 h and then irradiated with blue light. As shown in Figure 1E, while blue light increased Ca^2+^ influx through the TRPV1 channel, YCW extract reduced Ca^2+^ influx induced by blue light irradiation. This result indicates that YCW extract enhances cell proliferation decreased in blue light exposed-HaCaT cells and suggests its effect is mediated through downregulation of TRPV1 and inhibition of its phosphorylation.

### 3.2. Effects of Apigenin and Galangin on the Cytotoxicity of HaCaT Cells and the Proliferation of Blue Light-Irradiated HaCaT Cells

**Figure 2:**
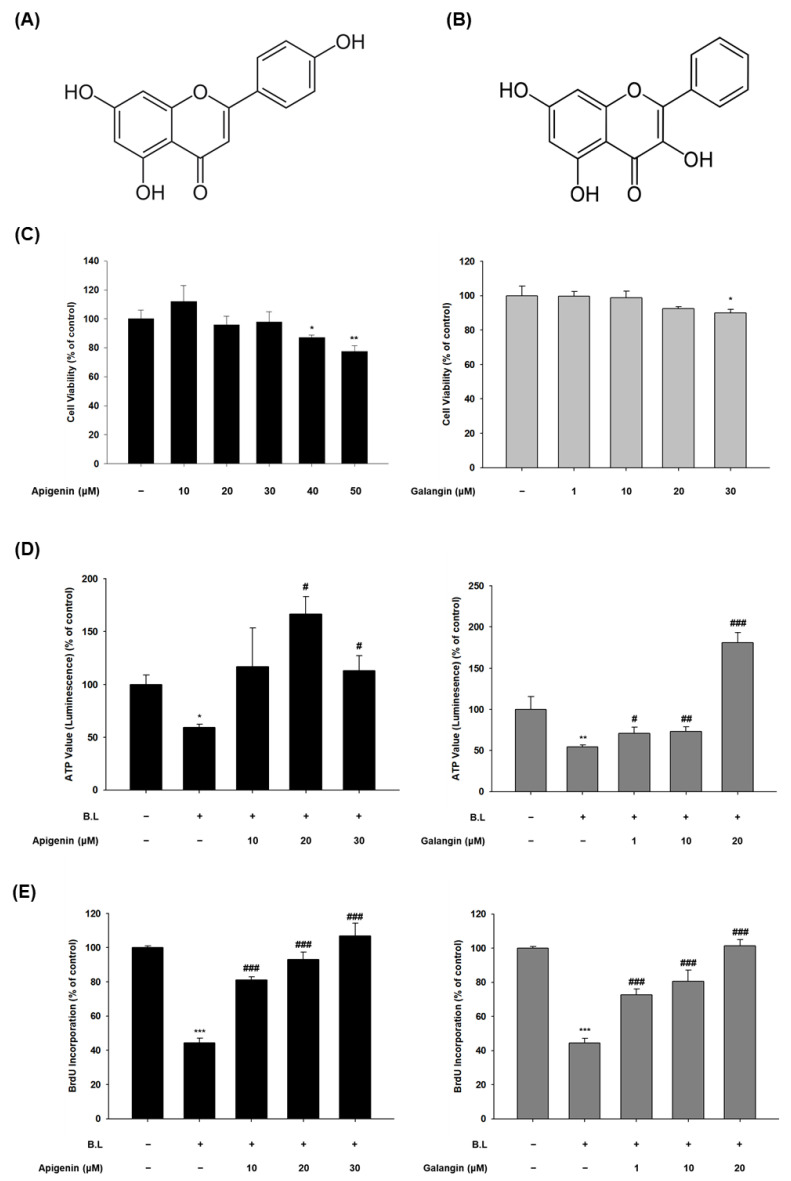

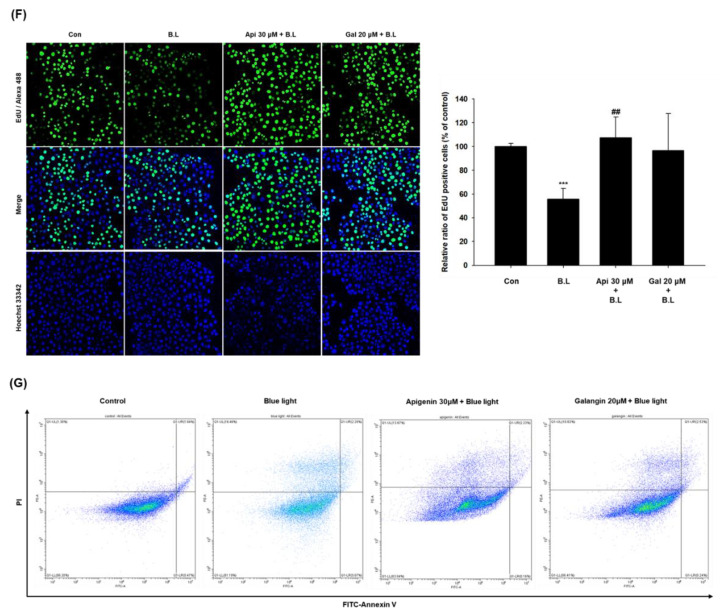
Apigenin and galangin, active components of YCW, increased proliferation in blue light-irradiated HaCaT cells. (**A**) Chemical structure of apigenin, (**B**) and galangin. (**C**) Cell cytotoxicity of apigenin (10–50 μM) and galangin (1–30 μM) were measured with CCK-8 assay. The effects of apigenin and galangin on proliferation of blue light (30 min, 76 W/m^2^)-irradiated HaCaT cells were determined by CellTiter-Glo^®^ 2.0 assay (**D**), BrdU ELISA assay (**E**), and EdU incorporation assay (**F**). Effect of apigenin (30 μM) or galangin (20 μM) on blue light-induced apoptosis in HaCaT cells was measured using flow cytometry analysis (**G**) * *p* < 0.05 vs. control, ** *p* < 0.01 vs. control, *** *p* < 0.005 vs. control, # *p* < 0.05 vs. B.L, ## *p* < 0.01 vs. B.L, ### *p* < 0.005 vs. B.L, V: viable cells, D: dead cells.

HaCaT cells were treated with different concentrations (10–50 μM) of apigenin (Figure 2A) and (1–30 μM) of galangin (Figure 2B) for 3 days and the percentage of cell viability was determined (Figure 2C,D). As shown in Figure 2C, apigenin was cytotoxic at 40 μM and the cell viability of galangin was decreased at 30 μM. Therefore, the maximum final treatment concentrations for apigenin and galangin were 30 and 20 μM, respectively. To evaluate the effect of apigenin and galangin on proliferation of blue light-exposed keratinocytes, the cells were pretreated before blue light irradiation with 10, 20, and 30 μM of apigenin or 1, 10, and 20 μM of galangin for 24 h. As shown in Figure 2D, both apigenin and galangin enhanced the cell proliferation reduced by blue light. In the BrdU ELISA assay to confirm the proliferation effect of apigenin and galangin, both apigenin and galangin significantly attenuated the inhibitory effect of blue light on proliferation of HaCaT cells concentration-dependently (Figure 2E). Similarly, EdU incorporation assay was conducted to prove cellular proliferation. As shown in Figure 2F, compared to the control group, blue light exposure remarkably reduced the population of EdU-positive cells. However, this effect was suppressed by pretreatment of apigenin (30 μM) and galangin (20 μM). In addition, this effect of apigenin and galangin was demonstrated using flow cytometry analysis after staining with FITC-annexin V and PI. Annexin V-stained group represents apoptotic cells, while PI staining is used to detect dead cells in a population. As shown in Figure 2G, the proportion of living cells in blue light-irradiated group reduced to 81.19% when compared to a non-irradiated group (96.20%). However, treatment with apigenin or galangin enhanced the ratio of living cells. Together, these indicate that blue light-irradiated reduction of cell proliferation is attenuated by either apigenin or galangin.

### 3.3. Apigenin and Galangin Suppress TRPV1-Mediated Signaling Induced by Blue Light-Irradiation

**Figure 3:**
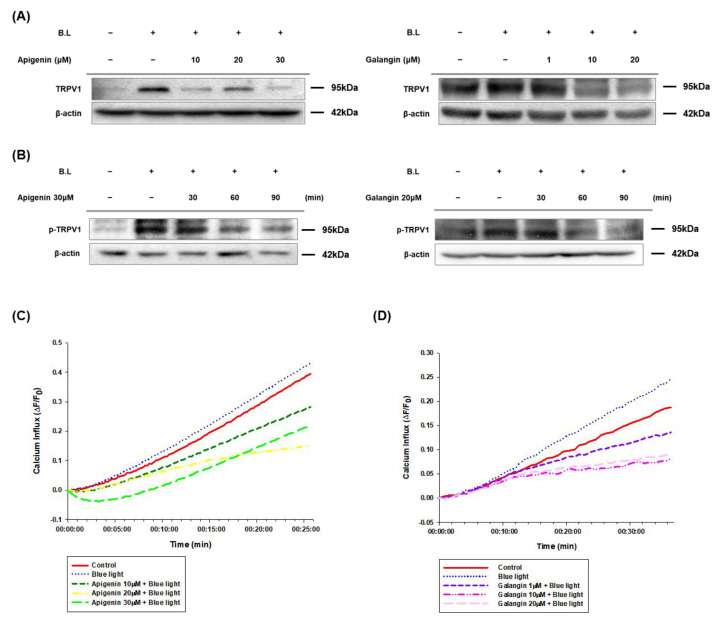
Apigenin and galangin reduced TRPV1 expression and its phosphorylation in blue light-irradiated HaCaT cells. (**A**,**B**) TRPV1 expression (**A**), and its phosphorylation levels (**B**) were determined by Western blotting. (**A**) Cells were incubated with apigenin (10, 20, 30 μM) and galangin (1, 10, 20 μM) for 24 h and were then irradiated with blue light (30 min, 76 W/m^2^). After 24 h incubation, the cells were subjected to the same process twice and were finally subjected to Western blot analysis. (**B**) Phosphorylation levels of TRPV1 were determined by Western blotting at 30-, 60-, and 90-min incubation after pretreatment with apigenin (30 μM) or galangin (20 μM) for 1 h and subsequent blue light-irradiation (30 min, 76 W/m^2^). The total proteins were extracted from the cells immediately after experimental conditions, and β-actin was used as a loading control. (**C**,**D**) Apigenin and galangin inhibited blue light-induced calcium influx in HaCaT cells. (**C**) Ca^2+^ influx changes of blue light and with apigenin (10, 20, 30 μM) pretreatment, (**D**) with galangin (1, 10, 20 μM) pretreatment by Fluo-4 NW assay. Cells were pretreated with apigenin (10, 20, 30 μM) and galangin (1, 10, 20 μM) for 1 h and irradiated with blue light (10 min, 76 W/m^2^). After the irradiation, fluorescence intensities were measured immediately over a certain period.

Previous studies in our laboratory have already shown that blue light upregulates TRPV1 and induce its phosphorylation [10]. Therefore, we examined effects of apigenin and galangin on blue light-induced upregulation of TRPV1. As shown in Figure 3A, various concentrations (10, 20, and 30 μM) of apigenin decreased the blue light-induced effect. Likewise, galangin also reduced the expression of TRPV1 induced by blue light irradiation (Figure 3A). In addition, TRPV1 phosphorylation contributes the activation of TRPV1 [52]. Therefore, we investigated effects of apigenin and galangin on TRPV1 phosphorylation. As shown in Figure 3B, blue light-induced phosphorylation of TRPV1 was reduced by both apigenin (30 μM) and galangin (20 μM). These data indicate that apigenin and galangin can antagonize against blue light effect by suppressing phosphorylation and expression of TRPV1.

Studies have shown that activation of the TRPV1 induces Ca^2+^ entry into the cytosol [51]. Therefore, we examined the involvement of apigenin and galangin in the blue light-induced calcium influx. As shown in Figure 3C,D, while blue light irradiation increased the inflow of Ca^2+^ compared to control group, apigenin and galangin reduced the calcium influx induced by blue light. These data indicate that apigenin and galangin antagonize TRPV1-mediated signaling induced by blue light irradiation.

### 3.4. Apigenin and Galangin Reduce ROS Generation in Blue Light Irradiated HaCaT Cells

**Figure 4:**
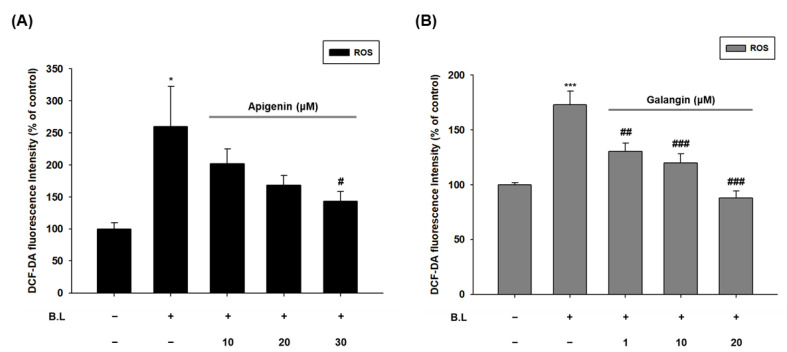
Apigenin and galangin decreased oxidative stress induced by blue light irradiation. (**A**,**B**) ROS production was determined by DCF-DA assay. Cells were incubated with apigenin (10, 20, 30 μM) (**A**) or galangin (1, 10, 20 μM) (**B**) for 24 h and were then irradiated with blue light (30 min, 76 W/m^2^). After 24 h incubation, the cells were subjected to the same process twice and were finally subjected to DCF-DA assay. * *p* < 0.05 vs. control, *** *p* < 0.005, # *p* < 0.05 vs. B.L, ## *p* < 0.01%, ### *p* < 0.005%.

Next, we examined whether pretreatment of apigenin or galangin ameliorates the accumulation of ROS in blue light irradiated HaCaT cells using a DCFDA dye. Treatment with apigenin or galangin significantly decreased endogenous ROS production induced by blue light irradiation in dose dependent manner, which was amplified by the blue light irradiation (Figure 4A,B). These data indicate that apigenin and galangin possess antioxidative activity.

### 3.5. Apigenin and Galangin Antagonize against Blue Light by Regulating MAPKs and MST-1/2-Akt-FoxO3a Signaling

**Figure 5:**
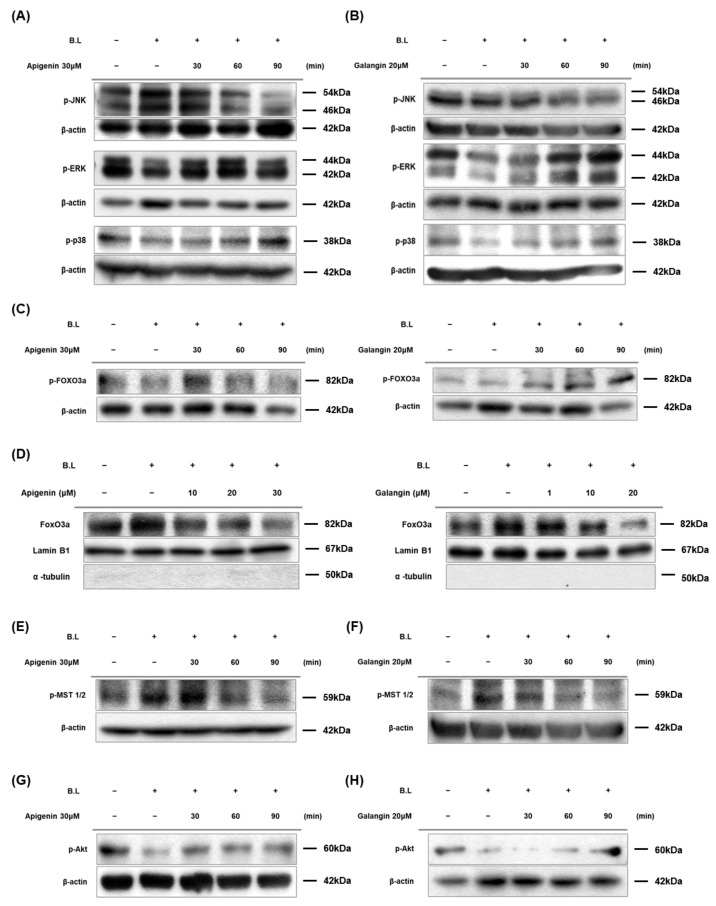
Apigenin and galangin antagonize against blue light by regulating MAPKs and MST-1/2-Akt-FoxO3a signaling. (**A**,**B**) Apigenin and galangin regulate phosphorylation of MAPKs in blue light-irradiated HaCaT cells. (**A**) Phosphorylation levels of MAPKs were determined by Western blotting at 30-, 60-, and 90-min incubation after, pretreatment with apigenin (30 μM) or galangin (20 μM) for 1 h and subsequent blue light irradiation (30 min, 76 W/m^2^). The total proteins were extracted from the cells immediately after experimental conditions, and β-actin was used as a loading control. (**C**,**D**) Apigenin and galangin inhibited FoxO3a nuclear translocation by FoxO3a phosphorylation. (**C**) Phosphorylation levels of FoxO3a were determined by Western blotting at 30-, 60-, and 90-min incubation after, pretreatment with apigenin (30 μM) or galangin (20 μM) for 1 h and subsequent blue light irradiation (30 min, 76 W/m^2^). The total proteins were extracted from the cells immediately after experimental conditions, and β-actin was used as a loading control. (**D**) The nuclear translocation of FoxO3a were determined by Western blotting at 24 h incubation after, 24 h apigenin (10, 20, 30 μM) and galangin (1, 10, 20 μM) pretreatment and 2-day repetitive blue light irradiation (30 min, 76 W/m^2^). The nuclear fraction was extracted from the cells immediately after experimental conditions by using NE-PER™ Nuclear and Cytoplasmic Extraction reagents. Lamin B1 was used as a loading control of nucleus fraction aliquot. (**E**,**F**) Apigenin and galangin suppress phosphorylation of MST-1/2. (**E**) Phosphorylation levels of MST-1/2 were determined by Western blotting at 30-, 60-, and 90-min incubation after, pretreatment with apigenin (30 μM) (**E**) or galangin (20 μM) (**F**) for 1 h and subsequent blue light irradiation (30 min, 76 W/m^2^). The total proteins were extracted from the cells immediately after experimental conditions, and β-actin was used as a loading control. (**G**,**H**) Apigenin and galangin activated Akt in blue light irradiated HaCaT cells. Phosphorylation levels of Akt were determined by Western blotting at 30-, 60-, and 90-min incubation after, pretreatment with apigenin (30 μM) (**G**), and galangin (20 μM) (**H**) for 1 h and subsequent blue light irradiation (30 min, 76 W/m^2^). The total proteins were extracted from the cells immediately after experimental conditions, and β-actin was used as a loading control.

Previous studies have reported that Ca^2+^ regulates MAPK functions [53]. Since apigenin and galangin were verified to lower Ca^2+^ flux by blue light irradiation, Western blot was performed to examine the effect of apigenin and galangin on MAPK activation. As shown in Figure 5A,B, apigenin and galangin attenuated the blue light effects on MAPKs. Specifically, apigenin and galangin reduced phosphorylation levels of JNK induced by blue light. On the contrary, while blue light reduced phosphorylation levels of ERK and p38 MAPK, this blue light effect was suppressed by apigenin and galangin treatment (Figure 5A,B). These results suggest that apigenin and galangin antagonize blue light effect by regulating MAPKs.

To investigate the role of FoxO3a, which is a downstream signal of MAPKs and regulator of cell proliferation, we examined effects of apigenin and galangin on phosphorylation levels and nuclear translocation of FoxO3a under blue light-irradiated condition. As shown in Figure 5C, while blue light irradiation reduced the levels of phosphorylated FoxO3a (Ser253), apigenin and galangin increased its phosphorylation levels. Additionally, FoxO3a was detected at higher level in the nuclear fractions of blue light irradiated group (Figure 5D). In contrast, apigenin and galangin treatment reduced the nuclear translocation of FoxO3a (Figure 5D). These results indicate that apigenin and galangin reduce nuclear translocation of FoxO3a by phosphorylating FoxO3a.

Mst-1/2 is known to phosphorylate FoxO3a and dissociate FoxO3a from 14-3-3 protein, which lead to nuclear translocation of FoxO3a [54]. To examine effect of apigenin and galangin on MST-1/2 activation, Western blot analysis for phosphorylated MST-1/2 was carried out. As shown in Figure 5E,F, while blue light irradiation caused significant increase in phosphorylated MST-1/2, apigenin and galangin reduced its phosphorylation levels. This data indicates that apigenin and galangin regulate MST-1/2- FoxO3a signaling. Furthermore, to investigate relationship between Akt and both FoxO3a and MST-1/2 signaling, we performed Western blot analysis for phosphorylation forms of them. As shown in Figure 5G,H, while blue light decreased the phosphorylation levels of Akt, apigenin and galangin induced the phosphorylation of Akt. These results indicate that apigenin and galangin antagonize against blue light by regulating MST-1/2-Akt-FoxO3a signaling.

### 3.6. Apigenin and Galangin Regulate Expression of Clusterin, Bax, and Bcl-2

**Figure 6:**
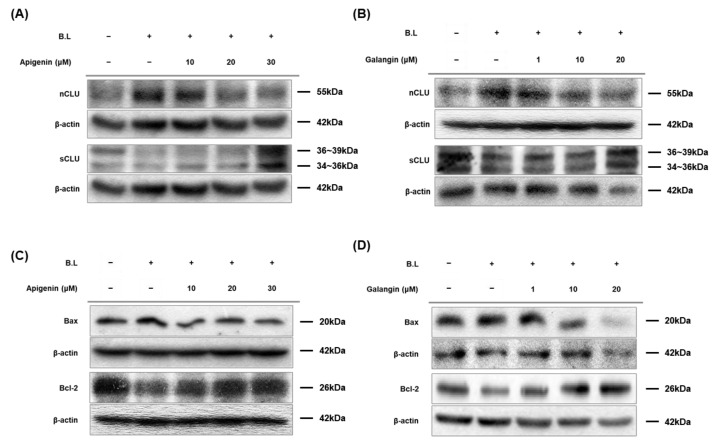
Apigenin and galangin regulated expression of nuclear and secretory clusterin as well as Bax/Bcl-2 ratio in blue light-irradiated HaCaT cells. (**A**,**B**) Protein levels of nuclear clusterin (nCLU) and secretory clusterin (sCLU) were determined by Western blot analysis. Cells were incubated with apigenin (10, 20, 30 μM) (**A**) or galangin (1, 10, 20 μM) (**B**) for 24 h and were then irradiated with blue light (30 min, 76 W/m^2^). After 24 h incubation, the cells were subjected to the same process twice and were finally subjected to Western blot analysis. The total proteins were extracted from the cells immediately after experimental conditions, and β-actin was used as a loading control. (**C**,**D**) Protein levels of Bax, apoptotic protein, and Bcl-2, anti-apoptotic protein, were determined by Western blotting. Cells were incubated with apigenin (10, 20, 30 μM) (**C**) or galangin (1, 10, 20 μM) (**D**) for 24 h and were then irradiated with blue light (30 min, 76 W/m^2^). After 24 h incubation, the cells were subjected to the same process twice and were finally subjected to Western blot analysis. The total proteins were extracted from the cells immediately after experimental conditions, and β-actin was used as a loading control.

Clusterin, Bcl-2 associated X protein (Bax), and B-cell lymphoma-2 (Bcl-2) have been well known to be involved in cell survival and apoptosis. We examined whether apigenin and galangin affect blue light-induced expression of two clusterin isoforms (nuclear clusterin and secretory clusterin), Bax and Bcl-2. As shown in Figure 6A,B, while blue light irradiation increased the expression of nuclear clusterin (pro-apoptotic protein), protein levels of secretory clusterin (pro-survival protein) were decreased. However, apigenin and galangin decreased expression of nCLU and increased expression of sCLU. In addition, as shown in Figure 6C,D, blue light irradiation increased protein levels of Bax (apoptotic protein) and reduced protein levels of Bcl-2 (anti-apoptotic protein). These blue light effects on Bax and Bcl-2 was attenuated by apigenin and galangin. These data indicate that apigenin and galangin exert antagonizing activity by in-creasing expression of cell survival-related genes.

## 4. Discussion

In everyday life, a large amount of blue light is irradiated to our skin through not only sunlight, but also electronic devices. The blue light is well known to reduce cell proliferation and induce oxidative damage, which in turn could cause photoaging [13,55,56,57,58]. Nevertheless, little is known about the mechanism for cell damage caused by blue light. In addition, only few substances have been reported to prevent the adverse effects of blue light. In this study, yellow chaste weed (YCW), and its main components such as apigenin and galangin were found to exert anti-blue light effect through regulation of TRPV1-mediated signaling.

First, our results showed that the YCW (*Helichrysum arenarium*), an herbaceous perennial plant, enhanced cell proliferation of human keratinocytes decreased by blue light irradiation. Next, according to our previous study, cellular damage caused by blue light irradiation in human keratinocyte is due to TRPV1 regulation [10]. TRPV1 cation channel can be activated by various stimuli and ligands such as heat (>43 °C), acid, capsaicin, and other vanilloid compounds (e.g., endocannabinoid, anandamide) [59]. Moreover, many studies have elucidated that TRPV1 expression is related to photoaging of the skin by UV [60,61,62]. Similar to UV results in these studies, our report has also proved that the expression of TRPV1 was increased by blue light irradiation [10]. Likewise, in this paper, we found that the TRPV1 expression elevated by the blue light was downregulated by high concentration of YCW extract (Figure 1C). In addition, phosphorylation of TRPV1 mediate channel’s activation [63]. Our data showed that blue light enhanced responsiveness of TRPV1 and yellow chaste weed extract dephosphorylated TRPV1 (Figure 1D). Further, TRPV1 channel opening stimulates Ca^2+^ entry into cytosol [14]. Blue light exposure increased Ca^2+^ influx, which was suppressed by YCW extract (Figure 1E). These data suggest that YCW extract could antagonize negative effects of blue light irradiation.

Apigenin and galangin, main flavonoids of YCW, showed the same effects as YCW extract. Specifically, while blue light inhibited keratinocyte proliferation, this blue light effect was attenuated by apigenin and galangin (Figure 2). Apigenin and galangin reduced expression and phosphorylation of TRPV1 as well as intracellular Ca^2+^ influx induced by blue light irradiation (Figure 3). These data suggest that apigenin and galangin has anti-blue light activity by regulating TRPV1 signaling.

ROS are produced during normal intracellular activation and are associated with various biological processes, including cell differentiation, gene expression, and response to cytokines [64,65]. Therefore, maintaining homeostasis of these ROS is critical for cell growth and survival. Oxidative stress is increased due to the imbalance between the production of ROS and the antioxidant reaction to remove it [64,66]. Thus, ROS has so far been recognized as a substance that simply oxidizes proteins, DNA, and lipids, causing cell necrosis, but also plays an important role in certain cytokines or growth factors signaling as an essential second messenger within cells [67,68]. In our study, we demonstrated that YCW extract, apigenin, and galangin can reduce intracellular ROS levels increased by blue light irradiation, suggesting that they possess anti-oxidative activity (Figure 4).

In addition, under various external stimuli, such as oxidative stress, mitogen-activated protein kinases (MAPKs) are involved in cell growth, apoptosis, and differentiation. MAPK subfamily consists of c-Jun amino-terminal kinase (JNK), extracellular signal-regulated kinase (ERK), and p38 kinase, and regulates the expression of various genes by phosphorylating transcriptional regulatory factors [69,70,71]. Activated JNK stimulates an apoptotic signaling by regulating expression of p53-dependent genes, such as Bcl-2 associated X protein (Bax) and p53-upregulated modulator of apoptosis (PUMA) [72,73]. JNK can also induce apoptosis by suppressing BH3-only family of Bcl-2 proteins [74]. In this study, apigenin and galangin reduced levels of phosphorylated-JNK (p-JNK), which can lead to anti-apoptotic signal (Figure 5A,B). In addition, both ERK and p38 kinase are known to promote cell survival, proliferation, and development [75,76,77]. In this study, apigenin and galangin also increased phosphorylation of ERK and p38 kinase which were reduced by blue light irradiation (Figure 5A,B).

MAPKs are known to regulate FoxO3a phosphorylation [78,79,80]. For example, under stress condition, activated JNK suppresses Akt activities which dephosphorylate FoxO3a and promotes FoxO3a nuclear translocation [78]. Specifically, phosphorylated Ser473 Akt can phosphorylate FoxO3a at Ser 253, leading to FOXO3a export from nucleus to cytosol and decreases its transactivation activity [32,33,34]. In addition, MST-1/2 is a positive regulator of FoxO3a activity [54]. MST-1/2 activation translocate FoxO3a from cytosol to nucleus and increase its transcription. Akt and MST-1/2 signaling negatively crosslink each other, and suppression of either Akt or MST-1/2 inhibits cell proliferation [81,82,83]. Activated p-Akt moves into nucleus, which phosphorylates FOXO3a and release from DNA to lower transcription activities [84,85]. The p-FoxO3a is exported out of the nucleus by binding to the 14-3-3 protein and remains inactive in the cytosol [86]. The FoxO3a transcription factors in the nucleus regulate target genes related to cell death, cell cycle arrest and oxidative stress [27]. In this study, we found that apigenin and galangin reduced phosphorylation of MST-1/2, and increased phosphorylation of Akt, leading to phosphorylation of FoxO3a and its export from nucleus (Figure 5C–H).

**Figure 7:**
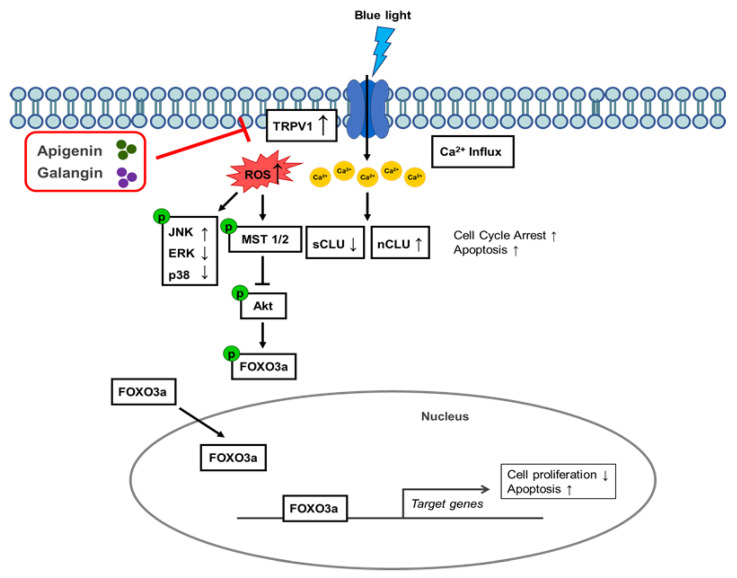
Schematic diagram showing action mechanism of apigenin and galangin in blue light-irradiated human keratinocytes.

Nuclear and secretory clusterin respectively carry out apoptotic or anti-apoptotic functions, by regulating Ku70, Bax, Bcl-2, and caspase activities [23,26,87,88,89]. In this study, blue light irradiation increased nuclear clusterin and reduced secretory clusterin, leading to reduced cell growth. Apigenin and galangin antagonized the blue light effect on expression of two isoforms of clusterin, consequently promoting cell proliferation (Figure 6). Collectively, these results indicate that apigenin and galangin exert anti-blue light effect by enhancing cell proliferation and decreasing ROS production through regulation of TRPV1 signaling and its related genes shown in Figure 7. However, since the connection between the molecules involved in the blue light-induced signaling was not clear, further study is needed to demonstrate it.

## 5. Conclusions

In conclusion, these findings indicate that apigenin and galangin, active components of YCW, can ameliorate negative effects of blue light through TRPV1/clusterin/FoxO3a and MAPK signaling pathways, suggesting the possibility of apigenin and galangin as an anti-blue light agent for human skin.

## Data Availability

The data used to support the findings of this study are available from the corresponding author upon request.
